# A Distinct Role of the Queen in Coordinated Workload and Soil Distribution in Eusocial Naked Mole-Rats

**DOI:** 10.1371/journal.pone.0044584

**Published:** 2012-09-05

**Authors:** Nobuyuki Kutsukake, Masayuki Inada, Shinsuke H. Sakamoto, Kazuo Okanoya

**Affiliations:** 1 Department of Evolutionary Studies of Biosystems, The Graduate University for Advanced Studies, Hayama, Japan; 2 Laboratory for Biolinguistics, RIKEN Brain Science Institute, Wako, Japan; 3 Precursory Research for Embryonic Science and Technology (PRESTO), Japan Science and Technology Agency, Kawaguchi, Japan; 4 Department of Cognitive and Behavioral Science, The University of Tokyo, Tokyo, Japan; University of Bristol, United Kingdom

## Abstract

We investigated how group members achieve collective decision-making, by considering individual intrinsic behavioural rules and behavioural mechanisms for maintaining social integration. Using a simulated burrow environment, we investigated the behavioural rules of coordinated workload for soil distribution in a eusocial mammal, the naked mole-rat (*Heterocephalus glaber*). We tested two predictions regarding a distinct role of the queen, a socially dominant individual in the caste system: the presence of a queen would increase the workload of other caste individuals, and the cues by a queen would affect the soil distribution. In experiment 1, we placed four individuals of various castes from the same colony into an experimental burrow. Workers exhibited the highest frequency of workload compared to other castes. The presence of a queen activated the workload by other individuals. Individuals showed a consistent workload in a particular direction so as to bias the soil distribution. These results suggest that individuals have a consensus on soil distribution and that the queen plays a distinct role. In experiment 2, we placed the odour of a queen in one of four cells and observed its effect on other individuals’ workload and soil distribution. Relative to other cells, individuals frequently dug in the queen cell so the amount of soil in the queen cell decreased. These results suggest that queen odour is an important cue in coordinated workload and soil distribution in this species.

## Introduction

In many group-living animals, individuals coordinate their activities and achieve collective decision-making to maintain social integration [1]–[7]. To identify the behavioural mechanisms involved in collective behaviour, it is necessary to investigate individual intrinsic behavioural rules [1]–[8]. It is also vital to consider variation in individual intrinsic factors such as sex, dominance rank, age, experience, or personality because different types of individuals might have different behavioural rules and might play a key role in collective decision-making [2], [5]–[7]. For example, a socially distinct individual (e.g., a dominant individual) occasionally, but not always, has a large impact on the consequence of collective decision-making [6], [7].

Nests with sophisticated architectures, which are excavated or constructed by the coordinated workload of group members, provide an interesting opportunity to study collective decision-making in group-living animals [1], [9]–[12]. A well-known example is nest construction or excavation in (eu)social insects (*e.g.* ants [13]–[17], bees [18], termites [19], and wasps [20]) in which individuals (mainly workers) follow simple behavioural rules and a coordinated workload [1], [10]–[12], [19]. The behavioural mechanism underlying such sophisticated coordination is composed of three components that are not mutually exclusive: template, stigmergy and self-organisation (reviewed in [1], [8]). The template indicates that the blueprint of a pattern exists in the environmental characteristics, such as physical or chemical heterogeneity. Stigmergy refers to an indirect interaction between an environmental cue and an individual, by which the individual behaves to reinforce the environmental cues. A “self-organised” pattern emerges as a result of positive, and often negative, feedback at the local scale, such as recruitment among workers. Previous studies in eusocial insects have shown through several examples that each mechanism, or a combination of mechanisms, operates during nest construction and excavation [1], [8], [12], [19], [20]. In species other than eusocial insects, however, the signals, cues, and information group members use and the behavioural rules that they have for coordinating nest excavation remain largely unexplored.

In this study, we experimentally investigated the coordinated workload and its effect on soil distribution in captive colonies of a eusocial subterranean mammal, the naked mole-rat *Heterocephalus glaber*. Naked mole-rats are one of only two vertebrate eusocial species [21]–[23]. Their colonies are composed of two to over 200 highly related colony members [22], [23]. The caste within a colony consists of one female (a queen), one to three reproductive males and many workers. In the wild, individuals dig a tunnel that ranges over several hundred metres [24]. Within the tunnel, individuals make spaces for different purposes, such as a nest chamber in which group members gather or rest, and a toilet chamber specialised for defecation [24]. Individuals dig and sweep unnecessary soil in a coordinated fashion from a nest [22]. The nest is important because colony members, including the queen, care for pups in the nest [25] and colony members must rest in the nest because of their poor thermoregulatory ability [22]. So, the collective nest excavation must be functionally important for this species in terms of maintaining social integration around the queen. Solitary species of subterranean rodents such as mole-rats of the family Bathyergidae also live in a structured tunnel in which places for different purposes are created (*e.g.* the nest chamber) [23], [26]–[29]. This suggests that group-living or eusociality is not necessary for such functional nest structure. It is reasonable to assume, however, that solitary and group-living species form those structures differently, because group-living species can coordinate workload and form longer and more complex structures than solitary species [30], [31].

In the present study, we conducted four different behavioural experiments (experiments 1 and 2 reported in the main text; experiments 3 and 4 reported in File S1, showing data that do not support the idea that initial soil distribution is reinforced by individual workload). Based on the eusocial system of this species, our study focused particularly on the role of the queen, a socially distinct and dominant individual. Several reasons exist to hypothesize that the queen has a strong impact on other individuals’ workload and soil distribution. First, a queen is the central individual in the colony, as she monopolises reproduction by reproductive suppression of workers [32], [33]. This is in contrast to the presence of plural reproductive males. A queen is the most aggressive individual with the higher level of testosterone than that of nonbreeding workers [34]–[36], which contrasts to less competition among reproductive males [37]. A queen may activate workload by others through aggressive behaviour ([34], [38], but see [36], [39] for non-supporting data). However, the role that the queen plays and the cues that colony members use for coordinating their workload have remained largely unknown.

In experiment 1, we first examined whether the workload by different individuals is coordinated, and tested a prediction that the presence of a queen specifically increases workload frequency by other caste individuals. If this prediction was supported, however, the design of experiment 1 did not determine whether this increase is by chemical (olfactory) or physical (*e.g*. direct contact) cues provided by the presence of a queen. These two cue types should have different effects since physical cues operate only during interaction and are generally paired with olfactory cues, while olfactory cues have long-lasting effects even when a queen is no longer present. In general, subterranean rodents depend on olfactory cues [40], which play an important social function in this species, such as colony member recognition [41], mate choice [42] and as a cue to detect the foraging route [43]. One study in naked mole-rats shows that individuals prefer their own colony odour to that of other colonies [41]. It remains unclear which individual’s odour strongly affects this odour discrimination as this study used mixtures of odours of individuals belonging to one colony. Furthermore, the function of odour on the workload except for foraging has not been investigated. Based on those backgrounds, we designed follow-up experiment 2 to examine the influences of a queen more specifically by placing the queen odour in one cell. We predicted that a cell with the queen odour would be emptied for the following reasons. First, if the queen odour activates the workload by other individuals, members should work most intensely in the cell with the queen odour. Second, because a queen rears pups in the nest chamber, one may reasonably predict that other individuals would prepare the nest chamber in the cell with the queen odour. By doing so, naked mole-rats may be able to achieve a consensus on the location of nest chambers and maintain social integration among colony members.

## Methods

### Ethics Statement

This study was conducted under the Japanese laws. The Wako Animal Experiment Committee of the Riken BSI approved all research protocols (#H21-2-243).

### Study Animals

This study was conducted with captive colonies of naked mole-rats maintained in the Laboratory for Biolinguistics, Riken Brain Science Institute, Japan. The study population was composed of individuals originally caught in the wild (ages unknown, brought into this laboratory in 1999) and their first-generation offspring. We maintained the animals in an artificial burrow system that was composed of acrylic boxes and connecting tubes that varied in size and length. Wood shavings were supplied as bedding. The temperature and humidity of the room was maintained at 30±2°C and 60±15%, respectively. The room was dimly illuminated for 24 h. The bedding was changed, and animals were fed vegetables and supplemental nutrients every other day.

The study animals in experiment 1 included 31 identified individuals (three queens: mass, 52.3 g, 60.0 g and 60.6 g, mean, 57.3 g; five reproductive males: mass range, 46.0 g–56.8 g, mean, 50.8 g; 16 male workers and seven female workers: mass range, 20.8 g–40.1 g, mean, 30.7 g) chosen from three established colonies whose group size varied from 10 to 28. The gender of the workers was determined by molecular biological methods [44]. In the experiment 2, we used three queens and 21 other individuals (five reproductive males, 10 male workers and six female workers from three groups) from the three colonies used in experiment 1.

### Experimental Design

Unless otherwise noted, experimental design was unchanged between experiment 1 and 2.

**Figure 1:**
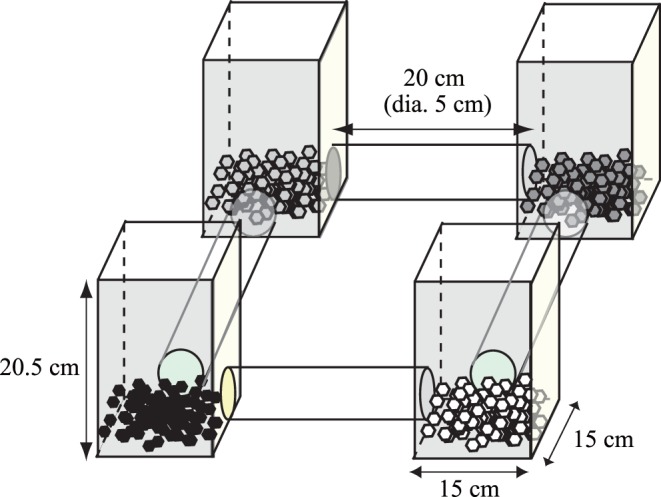
Experimental setup. Four cells were connected by tunnels in a circle. In total, 300 ‘soil’ particles of different colours were placed in each cell at the beginning of the experiment.

We conducted experiments lasting for 90 min in the room in which the animals were maintained during 13:00 to 17:00, during which they are usually active. We connected four acryl cells in a circle (15 cm × 15 cm × 20.5 cm height) using acryl tubes (diameter, 5 cm; length, 20 cm; Fig. 1). Three hundred soil-like materials (hereafter ‘soil’), which were made using commercially available drinking straws cut to 1 cm, were placed in each cell. This soil was approximately 1.5 cm high in the cells. By placing soils of different colours in each cell, we could compare the soil distribution before and after the experiments and estimate soil movement. Because of the degenerated vision capacity of the mole-rat, no reason exists that colour would affect the outcome. Although previous studies that investigated soil movement used real soils or chips [30], [45], [46], we did not use those materials because they are not easily quantifiable, and more importantly, because of potentially hygienic concerns for maintaining this species at our research institute.

We selected four individuals from the same colony and released them into the experimental space. To investigate the influence of each caste on other individuals’ behaviour, we randomised the individuals among experimental trials so as to make various combinations of four individuals (all castes in experiment 1; reproductive males and workers in experiment 2, see Table 1). The cell in which we released individuals was chosen randomly and did not affect the amount of soil found after the experimental session (data not shown). To facilitate identification, we marked the back of each individual with a different colour of oil-based paint (POSCA Mitsubishi pencil). This does not affect the animals’ behaviour since no self-grooming was observed. To avoid human disturbance, the experimenter left the room during the experiments and videotaped all behaviours for 90 min using a video camera 1 m above the experimental space. Typically, individuals circulated within the experimental space and started working after several minutes. Because of the small space, each individual was able to gain information on soil distribution and the behaviour of other individuals. At the end of the experiment (*i.e.* 90 min later), we removed the four individuals from the experimental space. We counted the number of different colours of soil in each cell. Although previous studies in rodents reported the magnetic compass orientation in building nests (e.g. [47]–[49]), such an effect has not been investigated in the naked mole-rat. In this experiment, the cell location did not affect the final soil distribution (linear mixed model: *F*_3,75_ = 0.91, *p* = 0.44). After the experiment, all cells, tunnels and soils were repeatedly washed, sterilised and deodorised (at least for humans) using disinfectant for the next experiment.

**Table 1:** The number of experiments for each pattern of composition.

colony	# queen	# reproductive males	# worker		exp 1	exp 2
I	1	0	3		3	–
	1	1	2		6	–
	0	1	3		3	6
	0	0	4		6	2
				Colony total	18	8
II	1	0	3		2	–
	1	1	2		4	–
	1	2	1		2	–
	0	1	3		4	4
	0	2	2		2	3
	0	0	4		5	5
				Colony total	19	12
III	1	0	3		3	–
	1	1	2		1	–
	1	2	1		2	–
	0	1	3		4	8
	0	2	2		2	2
	0	0	4		1	4
				Colony total	13	14
				total	50	34

Three colonies (I, II, III) are separately shown.

In Experiment 2, we placed the queen odour in one randomly chosen cell by putting the queen in the cell for 10 min prior to the experiment. The queen was then removed and an equal (300) number of soils was placed in each cell. Note that the soils did not contain the odour of the queen. This experimental design does differentiate which chemical component of the queen odour (scent from the skin or scent from excretory organs) is important for soil distribution, but our main purpose was to investigate the effect of natural vestiges of a queen on other individuals’ workload and soil distribution, not to isolate or evaluate which scent sources are important.

**Figure 2:**
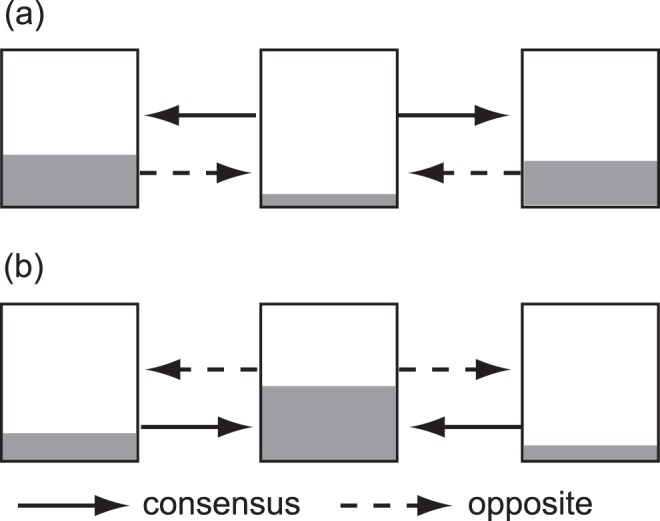
Illustrated explanation of our definition of “consensus” and “opposite” sweeping. (a) emptying *vs*. filling an emptied cell; (b) emptying *vs.* filling a filled cell.

In total, we conducted 50 and 34 trials with the mean number of experiments for each individual being 6.3 (range, 5–11) and 6.29 (range, 6–7) for experiment 1 and 2 respectively.

### Behavioural Definition

We coded two behaviours of workload used for nest excavation with different functions: digging behaviours in each cell ([22]; continuous activities of gnawing on hard cell walls, back-shovelling and foreleg digging lasting more than 5 s; *n* = 6231 and 5644 bouts for experiment 1 and 2); and sweeping behaviours between two cells ([22]; behaviour consisted of an individual kicking the straws behind itself while moving backward toward the neighbouring cell; *n = *5094 and 11,855 bouts for experiment 1 and 2). These two behaviours have different functions. Digging behaviour can be regarded as general workload to expand tunnels and the space in nest chambers. Sweeping behaviour functions to relocate unnecessary soil and empty or fill cells within a tunnel, and thus can be regarded as more “goal-oriented” for nest organisation. Although these two forms of behaviour can be clearly defined by human observers, the consequences of these two forms of behaviour, in terms of soil movement, may occasionally be inseparable as (i) individuals sometimes engage in these two behaviours successively (digging a cell soon followed by sweeping soil to a neighboring cell), and (ii) it is not rare that soil moves to a neighbouring cell as a result of digging.

**Figure 3:**
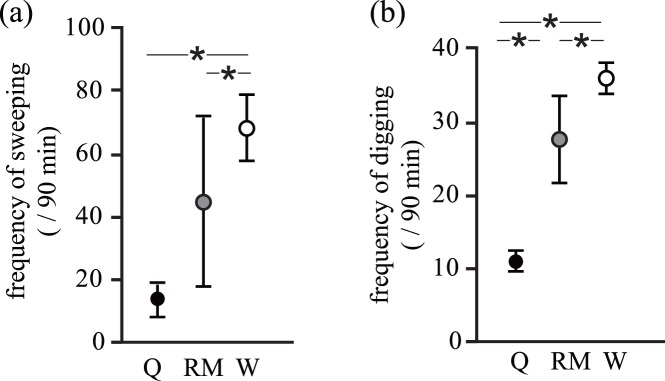
The total frequency of (a) sweeping and (b) digging behaviours during a 90 minute experiment. Results shown are for each caste in different experimental settings (Q: a queen, n = 3; RM: reproductive males, *n* = 5; and W: workers, *n* = 23). Asterisk indicates a significant difference. Individual mean ±1 S.E. is shown.

### Data Analyses

We used separate general linear mixed models (LMMs, *lme* function in *R* 2.13.1) or generalised linear mixed models (GLMMs, *lmer* function in *R* 2.13.1) to examine predictors of each dependent variable. Random terms were considered for repeated sampling. When necessary, we included the colony, the identity of the individuals and the observation date as random terms. We included all likely independent terms and a two-way interaction in the maximal model and sequentially excluded terms until the model included only significant terms. The alpha level was set at 0.05.

**Figure 4:**
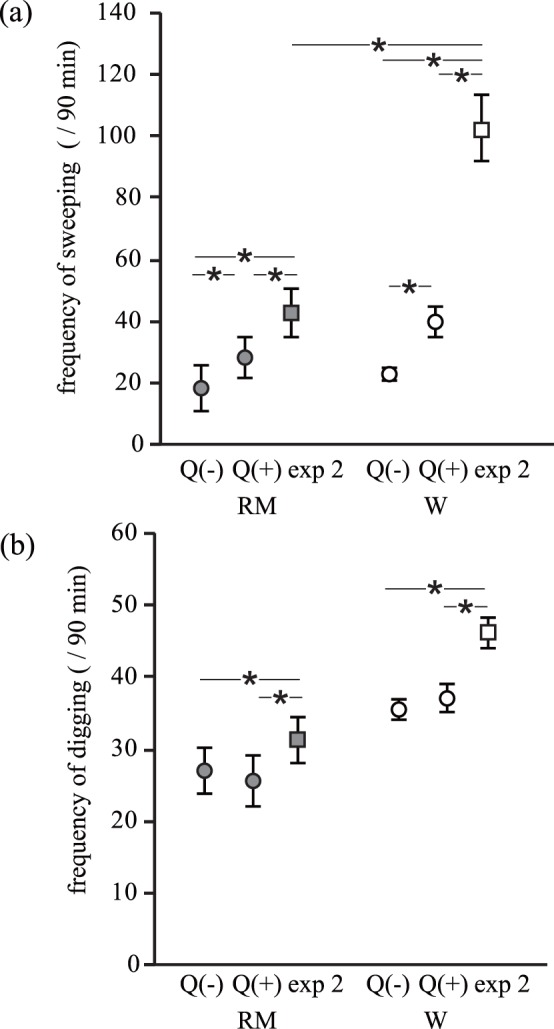
The total frequency of (*a*) sweeping and (*b*) digging behaviours during a 90 minute experiment. Results shown are for each caste (RM: reproductive males and W: workers) in different experimental settings. Q(–): experiment 1 without a queen (RM: *n = *5; W: *n* = 21); Q(+): experiment 1 with a queen (RM: *n* = 5; W: *n* = 23); exp 2: experiment 2 (RM: *n* = 5; W: *n* = 16). Asterisk indicates a significant difference. Individual mean ±1 S.E. is shown.

### Workload and Soil Distribution (Experiments 1 and 2)

If workload shaped the soil distribution, the workload at each cell and the number of soils should be associated negatively. We investigated the relationship between workload and soil distribution by investigating whether the workload at each cell was related to the distribution of soils after the experiment. We used LMM to investigate the relationship between the total frequency of workload in each cell (set as an independent term) and the total number of soils found in that cell after the experiment (irrespective of soil colour) or the number of soils found in the original cell after the experiment (quantified by considering soil colour, set as a dependent term).

**Table 2:** The effect of presence of a queen on workload (sweeping, n = 4902; digging, n = 5970) by other caste individuals in experiment 1.

	Sweeping			Digging		
	?^2^	df	p	?^2^	Df	p
Two-way interactions	52.24	1	<0.001	0.720	1	0.396
main effects	*b* (SE)	*z*	p	*b* (SE)	*z*	p
Caste (W > RM)	0.443 (0.285)	1.553	0.12	0.360 (0.148)	2.43	0.02
The presence of a queen (presence > absence)	0.139 (0.065)	2.129	0.03	0.054 (0.028)	1.94	0.052

RM: reproductive males. W: workers. The identity of colony (n = 3) and individual (n = 28) were determined at random. (See Table 1 for the number of trials in each colony.).

### Influence of Queen Odour on Soil Movement (Experiment 2)

To test a prediction that the soil in a cell with queen odour would be reduced in experiment 2, we compared the number of soil in the queen cell to that in other cells by LMM. Similarly, we considered the soil colour and compared the number of soil being carried away from the queen cell to that from other cells by LMM.

### Caste-Related Variation in Workload Frequency (Experiments 1 and 2)

Due to the eusocial system in this species, caste-related differences (i.e. workers > other castes) in workload were expected [15], [30]. To investigate the effect of caste on individual workload in both experiment 1 and 2, we ran a GLMM (Poisson error structure) in which each workload (the frequencies of sweeping or digging) was set as dependent term with caste as an independent term.

**Table 3:** Factors affecting sweeping behaviour in the context of emptying (n = 2580) or filling (n = 2613) cells in experiment 1.

	Emptying			Filling		
	?^2^	df	p	?^2^	Df	p
Two-way interactions	37.664	1	<0.0001	36.788	1	<0.0001
Frequency difference betweenconsensus minus opposite	*b* (SE)	*z*	p	*b* (SE)	*z*	p
Q < RM	1.091 (0.237)	4.617	<0.001	1.036 (0.214)	4.836	<0.001
Q < W	1.290 (0.223)	5.781	<0.001	1.189 (0.202)	5.864	<0.001
W = RM	0.199 (0.227)	0.892	0.764	0.153 (0.214)	0.757	0.762
Main effects						
Direction of the workload(consensus > opposite)	0.431 (0.092)	4.702	<0.001	0.501 (0.084)	5.941	<0.001
Caste						
Q < RM	0.518 (0.256)	2.023	0.0430	0.520 (0.184)	2.824	0.005
Q < W	0.438 (0.256)	1.711	0.04	0.707 (0.165)	4.284	<0.001
W = RM	0.080 (0.161)	0.501	0.617	0.186 (0.115)	4.273	0.105

Q: queen. RM: reproductive males. W: workers. The identities of colony (n = 3) and individual (n = 31) were determined at random. (See Table 1 for the number of trials in each colony.).

**Figure 5:**
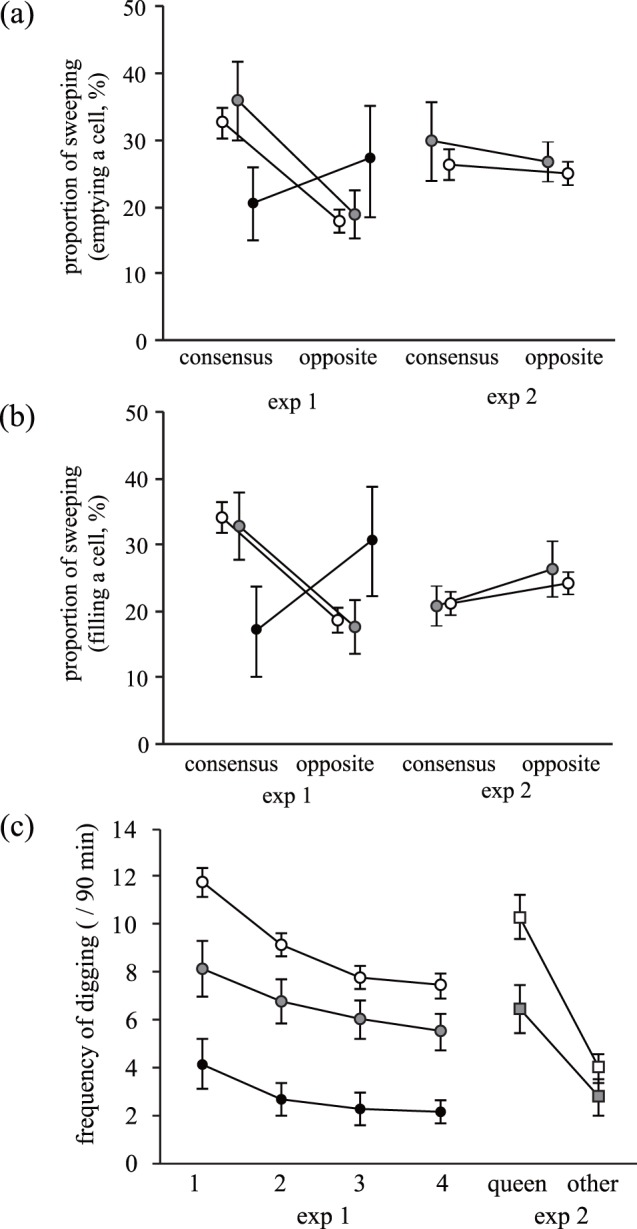
Relationships between workload and the type of workload (consensus building or opposite). Results given are by each caste category (black circles: queen; grey circles: reproductive males; white circles: workers) in experiments 1 and 2. (a, b) In experiment 1, the proportion of consensus building (a: removing soils from the emptied cell vs. its opposite; b: carrying soils into a filled cell vs. its opposite) of the total frequency of sweeping behaviour is shown (number of individuals: queen, n = 3; reproductive males, n = 5; and workers, n = 23). In experiment 2, the proportion of consensus building (removing soils from a queen cell vs. its opposite; number of individuals: reproductive males, n = 5; and workers, n = 16). (c) The frequency of digging in each cell. In experiment 1, the number on the x-axis indicates the order of cells according to the number of soils after the experiments (1 indicates a cell with the least number of soils, and 4 denotes a cell with the largest number of soils). In experiment 2, ‘queen’ and ‘other’ indicate a cell with the queen odour and other cells, respectively. Asterisk indicates a significant difference (p<0.05). Individual mean ±1 S.E. is shown.

### The Effect of a Queen Presence on Workload by other Members (Experiment 1)

In experiment 1, we specifically investigated whether the presence of a particular caste member (*e.g.* a queen) activated the workload of other individuals. We ran separate GLMMs (Poisson error structure) and examined how the presence of the queen (0 *vs.* 1) or the number of reproductive males (continuous variable: comparison among 0, 1 and 2) affected the workload of other individuals in each caste.

Comparison of the results between experiments 1 and 2 will elucidate the influences of queen odour, queen presence and the absence of a queen. Using a *post hoc* comparison using GLMMs, we investigated how the workload difference between workers and reproductive males was affected by the queen odour.

### Workload and Consensus (Experiments 1 and 2)

If the workload were coordinated, different individuals should behave in the same direction consistently during the experiments (consensus). To test this idea, we investigated whether the workload direction was consistent among individuals. In experiment 1, we first labelled the cells that had the largest number of soil after the experiment (range, 350–745) as a “filled cell”, whereas a cell that contained the least number of soils was called an “emptied cell” (range, 33–271). Next, sweeping for emptying an emptied cell (*vs*. filling an emptied cell; Fig. 2a), and filling a filled cell (*vs.* emptying a filled cell) was defined as consensus building (and as consensus breaking; hereafter referred to as “opposite”; Fig. 2b). Other sweeping behaviours were not considered in this study. In experiment 2, (1) digging of the queen cell was considered as consensus, whereas digging of another cell was classified as other; (2) sweeping behaviours with soil movement from the queen cell to a neighbouring cell was considered consensus, whereas movement of soil from neighbouring cells to the queen cell was considered the opposite.

Using a GLMM with a Poisson error structure, we compared the frequencies of workload for consensus building or opposite at the individual level as follows. First, we compared the frequency of consensus sweeping behaviour and its opposite both in experiment 1 (Fig. 2a,b) and experiment 2. Similarly, we compared the frequency of digging behaviours at each cell and investigated whether individuals dug following the order of cells arranged by the number of soils after the experiment using a GLMM with a Poisson error structure.

Another way to test consensus was to investigate whether the workload for building the consensus was consistent or increased with time. If the consensus was reinforced as the experiment progressed, the workload for consensus (*i.e*. to sweep soils from the emptied cell and to sweep soils into the filled cell; Fig. 2) was expected to increase with time from the start of the experiment. In contrast, if the workload for building a consensus were consistent throughout the experiment, no significant relationship was expected between the occurrence of consensus building and time during the experiment. We applied a separate GLMM with a binomial error structure with sweeping or digging behaviours (both, consensus *vs*. opposite; Fig. 2) set as dependent terms and the time from the onset of the experiment as an independent term.

Even if within-individual comparisons found that individuals worked in a consistent soil distribution direction more frequently than the opposite direction, this result may not necessarily mean that individuals have consistent workload throughout the experiment. One possibility is that individuals behave randomly during the early period of the experiment, and the consistent direction of workload might be aroused during the later period of experiments [1]. This possibility is problematic if the workload observed during the later period of the experiment determines the final soil distribution. This could affect which cells would be defined as filled and emptied. To test this possibility, we investigated the workload observed during the first 30 min of the experiment and tested whether a high frequency of consensus building could be observed during the early period of the experiment.

## Results

### Workload and Soil Distribution (Experiments 1 and 2)

In experiment 1, comparison among cells within an experimental trial showed that the total number of soils (irrespective of soil colour) in each cell was negatively correlated with the workload performed in each cell (sweeping: *b = *–2.194±0.509, *t*_192_ = –4.307, *p*<0.001; digging: *b* = –3.561±0.612, *t*_192_ = –5.821, *p*<0.001). In contrast, such relationships were not found in experiment 2 (digging: *b* = 0.661±0.518, *t*_98_ = 1.286, p = 0.201; sweeping: *b* = 0.048±0.163, *t*_98_ = 0.292, p = 0.771). In the analysis of soil by considering soil colour, the number of soils found in the original cell after the experiment was negatively correlated with the total amount of workload expended for each cell both in experiment 1 (sweeping: *b* = –1.899±0.186, *z* = –10.19, *p*<0.001; digging: *b* = –1.517±0.259, *z* = –5.85, *p*<0.001) and experiment 2 (digging: *b* = –1.373±0.175, *t*_98_ = –7.831, p<0.001; sweeping: *b* = –0.318±0.070, *t*_98_ = –4.554, p<0.001). These results indicate that the workload in each cell affected the number of soils in each cell, with fewer soils found in a cell with more frequent work effort.

### Influence of Queen Odour on Soil Movement (Experiment 2)

Queen odour affected the soil distribution in experiment 2, with a greater number of soils being carried away from the queen cell than from other cells. The number of soils after the experiment was less in the queen cell (mean ± S.E. = 261.58±18.73) than that in other cells (mean ± S.E. = 311.66±12.10; *b* = –50.08±23.59, *t*_78_ = –2.123, p = 0.034). An analysis of soil colour showed that the soils that were in the queen cell at the beginning of the experiment were found less often in the original cell (95.58±7.77) than ones that were initially put in other cells (117.00±5.24; *b* = –21.42±9.46, *t*_78_ = –2.265, p = 0.025).

### Caste-Related Variation in Workload Frequency (Experiments 1 and 2)

In experiment 1, the frequency of workload differed according to caste. Workers swept more frequently than reproductive males (*b* = 0.661±0.282, *z* = 2.339, *p* = 0.019) and the queen (*b* = 1.661±0.652, *z* = 2.548, *p* = 0.011; Fig. 3a). No difference was observed between the queen and reproductive males (sweeping: *b* = –1.00±0.69, *z* = –1.45, *p* = 0.15; Fig. 3a). Workers dug more frequently than adult males (*b = *0.36±0.14, *z* = 2.52, *p* = 0.01) and the queen (*b* = 1.196±0.241, *z* = 4.959, *p*<0.001; Fig. 3b). Moreover, the queen dug less frequently than reproductive males (*b* = –0.84±0.26, *z* = –3.17, *p* = 0.002; Fig. 3b).

In experiment 2, workers engaged in sweeping behaviours more frequently than reproductive males (reproductive males, 43.27±7.86, vs. workers, 103.5±10.80; *b* = 1.165±0.562, *z* = 2.073, p = 0.038; Fig. 4a), although no significant differences were observed in the total frequency of digging between reproductive males (31.5±3.21) and workers (46.26±2.07; *b* = 0.697±0.476, *z* = 1.463, p = 0.143; Fig. 4b).

### The Effect of a Queen on Workload by other Caste Members (Experiment 1)

In experiment 1, the variation in workload frequency among castes was affected by the presence of the queen. We found a significant two-way interaction between caste (reproductive males or workers) and the presence of a queen for sweeping frequency (Table 2; Fig. 4a). This result suggests that both reproductive males and workers increased their sweeping behaviour when a queen was present than when a queen was absent (Table 2; Fig. 4a), but the increase was more pronounced for workers than for reproductive males. Regarding the digging, no significant two-way interaction was observed between caste (reproductive males or workers) and the presence of a queen (Table 2; Fig. 4b). The presence of a queen increased the digging frequency by both reproductive males and workers, but this increase was not statistically significant (Table 2). Consistent with previous results, workers exhibited higher frequency of digging than reproductive males (Table 2).

In contrast, the number of reproductive males did not affect individual workload for each caste. The two-way interaction between caste and the number of reproductive males was not significant (digging: ÷^2^_2_ = 0.230, *p* = 0.631; sweeping: ÷^2^_2_ = 0.720, *p* = 0.392), and the main effects of the number of reproductive males had no statistical effect on the workload (digging: *b* = –0.078±0.061, *z* = –1. 279, *p* = 0.20; sweeping: *b* = –0.026±0.052, *z* = –0.487, *p* = 0.63). These results suggest that the presence of the queen specifically increased the workload by other individuals.

A comparison of the workload between experiment 1 and 2 showed that the variation caused by caste was more pronounced with queen odour. A significant two-way interaction was observed between the caste and type of experiment (χ^2^_2_ = 181.41, *p*<0.001). This result suggests that the difference in the frequency of sweeping behaviour between reproductive males and workers in experiment 2 was larger than that in experiment 1 with a queen (*b* = 0.623±0.054, *z* = 11.636, *p*<0.001) and that without a queen (*b* = 0.604±0.062, *z* = 9.794, *p*<0.001). The difference in the frequency of digging behaviour between reproductive males and workers did not vary among different types of experiments (two-way interaction: χ^2^_2_ = 3.603, *p* = 0.165). Individuals dug more frequently in experiment 2 than in experiment 1 with (*b = *0.177±0.025, *z* = 7.04, *p*<0.001) or without a queen (*b* = 0.209±0.021, *z* = 9.76, *p*<0.001; Fig. 4b).

### Workload and Consensus (Experiments 1 and 2)

In experiment 1, a comparison of the sweeping classified as consensus or opposite showed that the individuals had a consistent direction in their workload. However, two-way interactions between the types of sweeping and caste for both emptying and filling the cells (Table 3) suggest that the tendency to build consensus by each individual was different according to the caste. Consensus building was more frequent for sweeping into an empty cell than its opposite, but the effect varied among castes (Table 3); relative to the queen, both workers and reproductive males engaged in sweeping for consensus making more frequently than the opposite (Table 3; Fig. 5a). Similar results were found for sweeping to fill a cell (Table 3); consensus was more common than the opposite, and both workers and reproductive males engaged in sweeping for consensus more frequently than the opposite, relative to the queen (Table 3; Fig. 5b).

Each caste exhibited a similar digging pattern (Fig. 5c). The frequency of digging in each cell followed the decreasing order of soil number among the four cells (*b* = –0.157±0.011, *z = *–13.679, *p*<0.001), and this pattern was not different among castes (two-way interactions: χ^2^_2_ = 2.432, *p* = 0.2964).

An analysis of timing for the occurrence of consensus building suggested that the occurrence of sweeping for consensus building relative to the occurrence of sweeping in the opposite direction increased as the time of the experiments passed (emptying: *b* = 0.013±0.000, *z* = 4.691, *p*<0.001; collecting: *b* = 0.008±0.000, *z* = 2.905, *p* = 0.004). Relative to digging frequency in other cells, the frequency of digging behaviour in a filled cell decreased as time passed (*b* = –0.0001±0.0002, *z* = –6.398, *p*<0.001), whereas the digging frequency in an emptied cell did not vary through time (*b* = 0.0001±0.0002, *z* = –0.735, *p* = 0.462). These results suggest that individuals behaved in a consistent soil distribution direction. However, this result may have been caused by individuals behaving randomly and the workload observed in the latter period of the experiment affected the final soil distribution (see Methods). We found that this possibility is unlikely. The frequency for building a consensus during the first 30 min of sweeping behaviour was higher than that for the contrary workload (empty: *b* = 0.509±0.128, *z* = 3.976, *p*<0.001; filled: *b* = 0.754±0.074, *z* = 10.23, *p*<0.001). Furthermore, the frequency of digging behaviour at each cell during the first 30 min of the experiment followed the order of soil number after the experiment (*b* = –0.108±0.019, *z* = –5.706, *p*<0.001). These results suggest that the consensus building workload was consistently high at the early stage of the experiments and that its frequency increased as the experiment proceeded.

In experiment 2, the frequency with which group members dug the queen cell differed from digging frequencies in other cells (*b* = 0.069±0.030, *z* = 2.30, *p* = 0.02; Fig. 5c). However, the frequency of sweeping for consensus and the opposite did not differ (*b* = –0.062±3.239, *t*_254_ = –0.019, *p* = 0.940). The frequency of consensus building did not differ between workers and reproductive males (sweeping: *b* = –0.106±0.179, *z* = –0.595, p = 0.552; digging: *b* = 0.078±0.105, *z* = 0.739, *p* = 0.46). Relative to the occurrence of other workload behaviours, behaviours associated with consensus did not vary with time after onset of the experiment (sweeping: *b* = 0±0, *z* = –0.573, *p* = 0.566; digging: *b* = –0±0, *z* = –0.872, *p* = 0.383). In sum, these results suggest that individuals dug a queen cell more frequently than other cells and that this workload was consistent during the experimental periods.

## Discussion

The aim of this study was to reveal behavioural rules and the role of a queen during coordinated workload for soil distribution in eusocial naked mole-rats. In experiment 1, we found a complex interplay among the soil distribution and the workload contribution of each caste. A comparison of workload among castes showed that workers contributed most to soil distribution (Fig. 3). The result that workers showed a higher frequency of workload than reproductive males and the queen is a well-known pattern in naked mole-rats (division of labour [21] and [46]). This variation in workload among the castes was not straightforward; as predicted, the presence of a queen, but not the number of reproductive males, increased the frequency of sweeping behaviour by other castes, and this increase was particularly pronounced in workers (Fig. 4a). This result suggests that the presence of a queen facilitated coordinated soil movement. The presence of a queen also increased the frequency of digging behaviour, but the increase was not statistically significant (Table 2). Although how the queen activates the workload by other individuals remains unknown, one of the candidates is shoving behaviour (prolonged nose-to-nose pushing behaviour resulting in displacement of a subordinate individual; [34] and [46]). Whether shoving behaviour by a queen activates workers has met with both supporting [34], [38] and non-supporting data [36], [39]. However, in this study, shoving behaviour by the queen was observed only rarely (only three times in one colony).

In experiment 1, a consistent workload direction was found both in sweeping (Table 3; Fig. 5a,b) and in digging (Fig. 5c). Because these results were obtained from a mixed model, a consistent workload was confirmed at the individual level. One complication was the two-way interaction between caste and sweeping direction (for both emptying and filling), which suggests that a tendency to contribute to consensus building by the queen was weak relative to other castes (Table 3; Fig. 5a, b). Given that workload by the queen is relatively infrequent compared to that of other castes (Fig. 3), the effects of queen workload on the final soil distribution must be relatively small. However, this result suggests that the queen has a different behavioural pattern than that of other castes. We also found that the consistent sweeping direction for building a consensus and digging at an empty cell was evident even during the 30 min after the start of the experiments, indicating that individuals reached a consensus for workload direction at an early stage of the experiment. In other workload types whose frequency increased by time, social amplification among individuals or between individuals and environmental cues reinforcing the consensus might have occurred. This amplification process has been documented in nest excavation studies in eusocial insects [1], [17]. Workload could have produced a biased soil distribution, which becomes a cue for positive feedback by individuals to determine the workload direction. However, the results of experiments 3 and 4 (see File S1) showed that the initial bias in soil distribution was not reinforced after the workload, which does not support this idea. Therefore, one must consider an alternative hypothesis on how the coordinated workload was determined and amplified.

In experiment 2, we found that the queen’s odour was a sufficient cue to produce a biased soil distribution, with the number of soils in the queen’s cell being less than ones in other cells. The cell in which a queen was present was dug most frequently (Fig. 5c). The result that the consensus building workload did not vary according to time suggests inflexible behavioural rules. In contrast, the frequency of sweeping behaviour for consensus building was not different from that for the opposite direction, suggesting that the queen’s odour does not facilitate workload regarding soil movement for nest construction. This result is in contrast to the pattern found in the experiment 1 in which consistent sweeping behaviour for consensus building relative to that for an opposite direction was found. This difference suggests that the physical presence of a queen might be important for coordinated soil movement.

The odour of reproductive males may have similar effects. Although we did not conduct experiments in which the odour of other castes was marked, we suspect that the role of a reproductive male is not as special as that of the queen, and their influences are, if any, not a strong as those of a queen. First, the presence of reproductive males did not affect their workload in experiment 1. Second, only one queen is normally present in a colony of naked mole-rats but the number of reproductive males varies from one to three. Thus, reproductive males seem to be indistinct and do not control the queen (see also [35] and [37]).

Overall, the results of experiments 1 and 2 supported our two predictions that the queen has distinct influences on the workload undertaken by other colony members and on soil distribution. Consistent digging at a particular cell suggests behavioural rules of non-queen individuals to dig around the odour of a queen. This result seems reasonable given that the queen is a central individual in the naked mole-rat colony and the nest chamber is used for pup care and social integration [25]. Stigmergy or self-organisation may not be necessarily involved during soil distribution (see also File S1). The increase in consensus building workload found in experiment 1 appears to support the view that amplification occurs among individuals or between individuals and environmental factors. However, these results should be interpreted with caution because the placement of an olfactory cue by a queen, physical cue of a queen and behaviours of a queen were not fully controlled for in experiment 1. Although we do not deny the possibility that some forms of communication or recruitment were involved (e.g. recruitment among workers [43]), our conclusion, albeit slightly conservative, is that collective workload is based on an individual template. An example of a queen template has also been found in other eusocial species [50]–[52]. In one species of termites, *Macrotermes subhyalinus*, a pheromone released by the queen (or a freshly killed queen) induces worker construction of the royal chamber ([19]; Chapter 18 in [1]).

This study provides the first quantitative data on the role of a queen, and behavioural rules by other castes, in the context of the collective workload for soil distribution by the naked mole-rat. Based on our results, we feel to conclude that *all* behavioural rules were clarified is premature. For example, individuals in experiment 1 without a queen moved soils and built consensus comparable to that with a queen, suggesting that non-queen individuals follow some unrevealed behavioural rules to bias soil distribution and build a consensus. It is interesting to consider the possibility that individuals communicate during decision-making, which amplifies the consistency in workload along with queen odour. Since self-organisation processes based on the biased soil distribution was not observed (see File S1), other social mechanisms for workload coordination may exist. We could not analyse vocalisation during workload because it is impossible to identify the emitter of vocalisation when individuals are in a relatively small experimental setting with high levels of noise caused by workload. However, it might be that individuals used specific vocalization patterns to coordinate their activities as was found during the foraging [43]. Investigating whether similar behavioural rules operate in different experimental settings with different parameters or in a wild group is also important. Our experiments were conducted within artificial and narrow settings compared to the natural situation, and the number of individuals was fixed to four to avoid crowding. The “soil” used in this experiment, which was easily quantifiable, may be relatively lighter and less costly for soil movement compared to energetic costs in natural conditions [45], [53]–[56]. Those factors suggest that the results of this study should be treated with caution. However, we would like to emphasise that it is unlikely that the experimental setting affected the fundamental behavioural patterns of the naked mole-rat because part of the results regarding workload found in this study replicates findings in previous studies despite of different experimental settings (variation among caste [21], [46] and the inducing function of workload by a queen [34], [38]). It is perhaps preferable to investigate workload in different experimental settings by changing the morphology of experimental space along with the number of subjects. Changing those parameters would help clarify unrevealed behavioural rules and their flexibility. A limited number of studies are available on the way in which individuals coordinate their workload in wild naked mole-rats [24]. The results of our experiments suggest that a queen is the key individual for determining the workload frequency of other individuals and possibly tunnel morphology in the wild. To test this possibility in the wild, continuous recording of behaviour by the queen and others within a tunnel, as well as detailed data on temporal changes of tunnel morphology, are necessary. Given that the burrow in a natural population ranges widely over several hundred metres, to hold that the effect of a queen operates on all other colony members is not always true. We believe that the naked mole-rat is a good model system, both in captivity and in the wild, to investigate the behavioural rules and processes of collective workload.

## Supporting Information

File S1 **Results of experiments 3 and 4.**
(DOC)
