# Supplementary material for: A Distinct Role of the Queen in Coordinated Workload and Soil Distribution in Eusocial Naked Mole-Rats
Source: PLoS One. 2012 Sep 5;7(9):e44584. doi: 10.1371/journal.pone.0044584 (PMC3434143; doi:10.1371/journal.pone.0044584)
Supplement: File S1 — Results of experiments 3 and 4. (DOC) [file pone.0044584.s001.doc]

**Supporting Information**

We report the results of two additional experiments in which other types of behavioural rules were tested but were not supported. In experiment 1, we found that the workload for building consensus increased as time from the beginning of the experiment passed. This may have been caused by stigmergy and a self-organisation process by which each individual had simple behavioural rules to reinforce the existing soil distribution bias, and this bias was further reinforced by their workload. To investigate this possibility, we conducted two additional experiments in which the initial distribution of soils was changed and observed whether individuals reinforced this biased soil distribution level.

*Methods*

The fundamental design of the experiments was the same as that of experiment 1, except that the initial soil distribution was changed.

In experiment 3, we placed 100 soils in one cell and 300 soils in three other cells. This design tests the possibility that individuals reduce soils in a cell that has the fewest number of soils.

In experiment 4, we placed 300 soils in one cell and 100 soils in three other cells. This design enabled us to investigate whether individuals move soils to the cell with 300 soils.

We used all castes in these experiments (n = 26 and 24; mean number of experiments per an individual = 3.54 and 4.33, respectively), but the results of soil distribution did not differ according to the presence of a queen or reproductive males. In total, we conducted 23 trials (6, 7 and 10 for each colony) for experiment 3 and 26 trials (seven, nine and 10 for each colony) for experiment 4.

*Results and discussion*

In all experiments, except for one trial in experiment 4, the distribution of soils was not biased, but levelled after the experiments (experimental 3: three 100 rooms: 251.88 ± 11.72 vs. 300 room: 242.35 ± 16.99; experimental 4: three 300 rooms: 142.87 ± 6.28 vs. 300 room: 171.87 ± 11.48). In one exception, a cell with 100 soils initially was reduced to 51 soils.

These results indicate that the initial soil distribution was not reinforced and that the self-organisation process based on uneven soil distribution was not confirmed.
